# 121. Mucormycosis and COVID-19 in the United States: a Real-World Evidence Analysis of Risk Factors and Survival Among Patients with Mucormycosis, with and without COVID-19 Preceding the Infection

**DOI:** 10.1093/ofid/ofab466.121

**Published:** 2021-12-04

**Authors:** Kaylen Brzozowski

**Affiliations:** TriNetX, LLC, Cambridge, Massachusetts

## Abstract

**Background:**

Mucormycosis has been associated with COVID-19 infections, notably in India, and known risk factors for mucormycosis such as diabetes mellitus have been studied in this context. This analysis aims to characterize patients in the US with mucormycosis, with and without COVID-19, by risk factor and mortality.

**Methods:**

Data from the TriNetX Research Network representing over 66M de-identified patient-lives in the US was used to examine characteristics and outcomes among mucormycosis patients with and without preceding COVID-19 infection. Patients must have had a mucormycosis diagnosis recorded from 1/1/2020 to 6/8/2020. Patients were then identified as having either a COVID-19 diagnosis or positive SARS-CoV-2 RNA laboratory result (M+COV) or no COVID-19 diagnosis or positive RNA result (MnCOV) any time prior to through one day after the mucormycosis diagnosis. These cohorts were evaluated across characteristics recorded in the EMR within 1 year prior to and including the date of mucormycosis record. Mortality was evaluated with Kaplan-Meier statistics as survival until recorded death on or after mucormycosis diagnosis.

**Results:**

Of 302 patients with mucormycosis from 1/1/2020-6/8/2021, 30 patients (10%) had M+COV, and 272 (90%) had MnCOV. Among the M+COV cohort, 22 patients (73%) had mucormycosis recorded within 2 weeks of COVID-19 infection. The M+COV and MnCOV cohorts had majority male sex (60,59%;p=0.93) and a similar prevalence of transplanted organs (40,28%;p=0.16), long-term drug therapy (60,54%;p=0.56), chronic kidney disease (43,31%;p=0.16), and glucocorticoid treatment (67,64%;p=0.76). The M+COV cohort had a greater prevalence of type II diabetes mellitus (67,35%;p< 0.01), acidosis (53,22%;p< 0.01), and posthemorrhagic anemia (43,14%;p< 0.01) than the MnCOV cohort. M+COV patients seem to progress to mortality more quickly than MnCOV patients (p=0.01, see Figure 1).

Figure 1. Survival until all-cause mortality after mucormycosis diagnosis, 0-180 days, among patients with (M+COV) and without (MnCOV) COVID-19 preceding the infection.

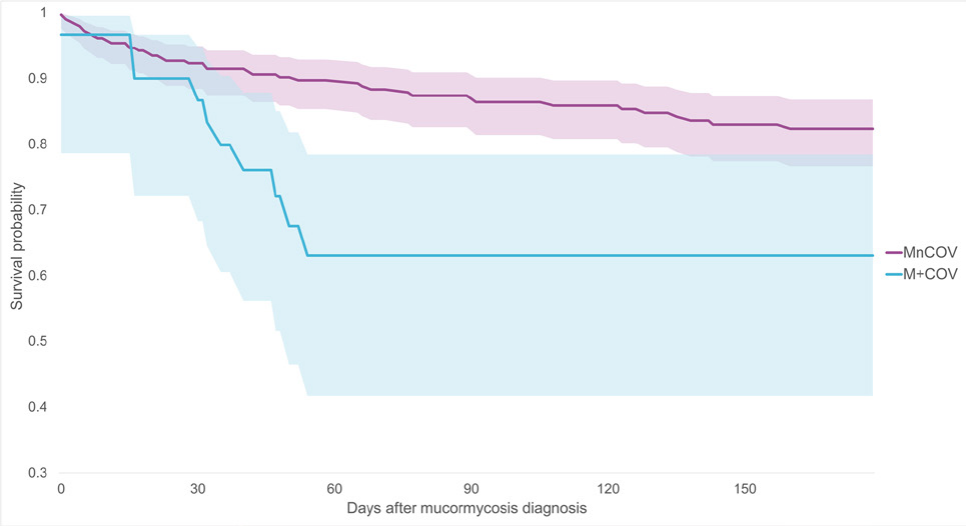

**Conclusion:**

This study found that patients in the US with mucormycosis and current or previous COVID-19 infection have a greater prevalence of underlying conditions, including diabetes, and more rapid progression to mortality than those without COVID-19. The nature of the potential relationship between comorbidities, mucormycosis, and COVID-19 should be explored further.

**Disclosures:**

**All Authors**: No reported disclosures

